# Experiencing COVID-19 symptoms without the disease: The role of nocebo in reporting of symptoms

**DOI:** 10.1177/14034948211018385

**Published:** 2021-05-27

**Authors:** Hojjat Daniali, Magne Arve Flaten

**Affiliations:** Department of Psychology, Norwegian University of Science and Technology (NTNU), Norway

**Keywords:** COVID-19, coronavirus, negative emotions, nocebo effect, psychological factors

## Abstract

**Background:**

A nocebo effect occurs when inactive factors lead to worsening of symptoms or reduce treatment outcomes. Believing that one is or has been infected with COVID-19 may act as a nocebo. However, not much is known about potential nocebo effects associated with the reporting of COVID-19 symptoms.

**Aim:**

An online survey investigated whether certainty of being infected with COVID-19, age, sex, cognitive, emotional and personality factors were associated with perceived severity of COVID-19 symptoms.

**Methods:**

Participants (*N*=375) filled out an online survey containing 57 questions asking about symptoms resembling COVID-19, certainty of being infected with COVID-19, anxiety, stress and personality dimensions.

**Results:**

Certainty of being infected with COVID-19 and anxiety predicted 27% of the variance in reporting of COVID-like symptoms. The mediation analysis showed that both higher certainty of being infected and anxiety independently predicted increased reports of COVID-like symptom. Females had higher anxiety and stress levels, and reported more COVID-like symptoms than males did. Older age was not associated with reporting COVID-like symptoms.

**Conclusions:**

Believing to be infected with COVID-19, along with anxiety, can enhance the severity of COVID-like symptoms. Thus, the nocebo effect was due to both cognitive and emotional factors and was higher in females.

## Introduction

A nocebo effect occurs when a medically inactive agent or intervention leads to worsening of symptoms or reduced treatment outcomes [1]. Negative expectations and/or prior experiences related to treatments (whether active or not) are known as the underlying causes of nocebo effects, which account for a substantial proportion of reporting of adverse side effects of medical treatment and increased pressure on the health service [2].

COVID-19 is a highly infectious pneumonia characterised by dry cough, fever, fatigue, bodily pain and shortness of breath. However, the severity of symptoms and the course of the disease is highly variable among individuals, ranging from asymptomatic carriers to patients in critical care. Based on the severity of symptoms, symptomatic patients are classified as mild, severe and critical cases. Mild patients have minor to no pneumonia. Severe patients have shortness of breath, a respiratory rate higher than 30 breaths per minute and blood oxygen saturation <93%. Critical patients have respiratory failure, septic shock and/or multiple organ dysfunction or failure. The time from onset of illness to shortness of breath is 8–10 days. Having coexisting medical conditions or being elderly is correlated with poorer treatment outcomes and higher mortality rates [3].

Outbreaks contribute to the development of physiological and psychological distress. During the Middle East Respiratory Syndrome-Corona Virus (MERS-CoV) outbreak, higher pandemic-related stress was correlated with higher general anxiety in females [4]. During the present pandemic, fear of COVID-19 is shown to be associated with depression, anxiety and health anxiety [5]. Being constantly informed about the pandemic and high exposure to social media related to COVID-19 [6] are also associated with anxiety and depression. The psychological distress due to the pandemic may also contribute to the development of health-related worries and concerns about the infection, as worries about health have been reported to increase during public pandemics (e.g. Xie et al. [7]).

The belief of being infected with COVID-19 may act as a nocebo and generate negative expectations about bodily sensations, resulting in perception of bodily sensations as COVID-19 symptoms. Such a nocebo, along with psychological processes such as anxiety and stress, could thus enhance the perception of bodily experiences such as minor pain, difficulty in breathing, coughing and so on as COVID-like symptoms.

Moreover, cognitive, emotional and personality factors (e.g. neuroticism [8]) have been shown to contribute to symptom reporting. Bailer et al. [9], for instance, reported that for participants with a specific type of somatoform disorder, multiple chemical sensitivity, a model consisting of cognitive (thoughts about environmental threats), emotive (general anxiety) and habitual traits (a tendency to focus more on bodily sensations) predicted more than one third of the variance for over-reporting (i.e. reporting more symptoms than what is being experienced, or misattribution of bodily experiences) of symptoms compared to non-somatoform participants. Similar models on the effects of cognitive, personality and emotive factors in response to COVID-19 pandemic have been tested, and characteristics such as health anxiety and personality traits such as neuroticism [10] have been reported to be associated with higher COVID-19 anxiety and to accelerate the development of health worries.

However, no study has yet explored whether the belief of being infected with COVID-19 can account for reporting of COVID-like symptoms. Therefore, we proposed a model where a cognitive process (certainty of being infected) predicts the reporting of COVID-like symptoms. Next, we investigated whether the effect of certainty of being infected on reporting of COVID-like symptoms was mediated by emotional and personality factors. As emotional factors have been shown to mediate placebo and nocebo effects [11], we hypothesised that higher anxiety and stress mediated the association of certainty of being infected with reporting of COVID-like symptoms. Furthermore, it was hypothesised that personality dimensions, specifically higher scores in neuroticism and conscientiousness (based on the five-factor model of personality), mediated the association of certainty of being infected on reporting of COVID-like symptoms. Fourth, COVID-19 more seriously affects older individuals, and older participants were hypothesised to report more symptoms, and age was assumed to moderate the effect of certainty on COVID-like symptoms. Finally, females were hypothesised to report more symptoms, and sex was also assumed as a moderating factor, as females were shown to have higher anxiety during MERS-CoV outbreak, and nocebo effects have been found to be greater in females [12].

## Methods

### Respondents

The respondents included 279 females (min_age_=16 years, max_age_=71 years, range=55 years, *M*_age_=32.9 years, standard deviation (*SD*)=10.4 years), 135 males (min_age_=17 years, max_age_=79 years, range=62 years, *M*_age_=33.2 years, *SD*=12 years) and three reporting as ‘other gender’ who completed an online anonymous survey from 2 May 2020 to 3 August 2020. Almost two thirds (64.5%) of the sample held an academic degree equivalent to a master’s or a PhD. Nearly all (90.6%; *n*=378) of the sample had not been tested for COVID-19. Of those who had been tested, only 1.4% (*n*=6) had tested positive, and 7.9% (*n*=33) had tested negative. Based on the aim of the study, which was investigating the predictive value of the belief certainty of being infected with COVID-19 for reporting of COVID-like symptoms, only male and female respondents who had not been tested for COVID-19 were included in the analyses. Therefore, a total sample size of 375 participants (min_age_=16 years, max_age_=79 years, range=63 years, *M*_age_=32.7 years, *SD*=10.9 years), including 249 females (min_age_=16 years, max_age_=67 years, range=51 years, *M*_age_=32.6 years, *SD*=10.2 years) and 126 males (min_age_=17 years, max_age_=79 years, range=62 years, *M*_age_=32.8 years, *SD*=12.1 years), were included in the analyses.

### Measures

#### COVID-19 symptoms

Respondents were asked to report the severity of a group of symptoms related to COVID-19 by rating 10 questions. Using a five-point Likert scale that ranged from 0=‘none’ to 4=‘severe’, respondents specified the severity of any fever, myalgia (bodily pain), headache, cough, dry cough, sore throat, difficulty in breathing, fatigue and persistent fever and the experience of fever, repeated dry cough and difficulty in breathing at the same time during the last two months prior to filling out the form. The total sum of the items was used in the analyses. Similar items have been used in prior studies to assess the physical symptoms of COVID-19 in the general population [13]. Cronbach’s alpha for COVID-19 questions in the present study was 0.85.

#### COVID-19 certainty

Respondents rated their certainty level of being infected with COVID-19 on a five-point Likert item ranging from 0=‘sure not infected’ to 4=‘certain that infected’.

#### Stress and anxiety

Stress and anxiety were measured using the short form of the Depression Anxiety Stress Scale (DASS-21) [14]. The DASS-21 has three subscales that each include seven items that are rated on a four-point Likert scale. The subscale scores are calculated by summing the scores for the seven items belonging to each subscale and multiplying by two. The DASS-21 has acceptable psychometric features and is used in different samples. To avoid fatigue effects due to large number of questions, only the stress and anxiety subscales were used in this study. In the present study, the internal consistency for the anxiety and stress subscales were 0.80 and 0.86, respectively.

#### Personality dimensions

The short version of the Big Five Inventory (BFI-10) [15] was administered to assess the personality dimensions. The BFI has 10 items and assesses the five-factor model of personality that includes extroversion, agreeableness, conscientiousness, neuroticism and openness. Two questions are dedicated to each personality dimension. The BFI-10 is scored on a five-point Likert scale ranging from 1 to 5. The BFI-10 is reported as a valid personality inventory with acceptable psychometric characteristics.

#### Demographic questions

Respondents answered questions asking about their sex, age, education level and the result, if relevant, of any COVID-19 test. The options for education were: ‘<10 years of school’, ‘10 years of school’, ‘high school degree (12–13 years school)’, ‘bachelor’ and ‘master’s/PhD or equivalent’. Health anxiety was also assessed with 18 items, but as it overlapped with anxiety, the data related to health anxiety are not analysed in this study. In total, the survey consisted of 57 items that were mostly Likert-type questions.

### Procedure

Using a snowball sampling strategy focused on the general population during COVID-19 pandemic, the online survey was first disseminated to the Norwegian University of Science and Technology (NTNU) students and staff through the intranet, and then the link to the survey was shared via social media applications, including Facebook, Instagram, Twitter and LinkedIn. Both COVID-19 patients and healthy individuals were invited, since the goal was testing the hypotheses in the general population. Respondents were assured that no personal data would be recorded in this survey. Before filling out the questionnaires, respondents provided informed consent to be included in the study. In the consent form, participants were informed that the study investigated ‘the effects of psychological factors on symptoms related to COVID-19’, and they should respond to questions asking about their thoughts, personality, stress, anxiety and some physical symptoms that could be related to COVID-19. The inclusion criteria were being aged 16 years or above and being able to read English, as the survey was in English. The study was approved by the Regional Committee for Medical and Health Research Ethics (REK; project number: 142652) and the Norwegian Centre for Research Data (NSD; project number: 605612).

### Statistical analyses

The data were analysed with IBM SPSS for Statistics v27 (IBM Corp., Armonk, NY) and by the following steps. First, outlier data were detected and resolved. Then, the descriptive statistics were investigated. Next, the correlations between the variables were investigated across males and females using Pearson two-tailed correlation. Then, the assumptions for conducting a multiple linear regression were tested (see the next section). Next, a moderated mediation analysis was tested using the PROCESS macro for SPSS [16] (applying model 8 with 5000 bootstrap samples). Due to the non-normality of the residuals (see the next section, Data Screening and Pre-processing), the Huber–White heteroscedasticity-consistent (HC) standard errors were used. In the moderated mediation analysis model, only variables that correlated with the report of COVID-like symptoms were entered. Then, the significant mediation effects were tested by a Sobel test, following the recommendations of Baron and Kenny [17]. Lastly, a trend analysis, using a two-way analysis of variance (ANOVA), was performed to investigate differences in COVID-like symptoms between age groups. Moreover, another ANOVA was performed to investigate differences in anxiety and stress between males and females.

### Data screening and pre-processing

First, the outliers were resolved using the Winsorising technique. Next, to test for the basic assumptions of a regression (i.e. normality, heteroscedasticity, homoscedasticity, multicollinearity and linearity), a multiple regression was run. The total sum of the COVID-like symptoms was entered as the dependent variable (DV), and age, sex, certainty of being infected, stress, anxiety and five personality factors were entered as independent variables (IVs). The results showed that sex (*B*=−1.31, *SE*=0.51, β=−0.11, *p*=0.01), certainty of being infected (*B*=1.40, *SE*=0.25, β=0.25, *p*=0.0001) and anxiety (*B*=0.28, *SE*=0.05, β=0.38, *p*=0.0001) predicted the DV. However, the histogram plot for residuals suggested a non-normal distribution of residuals, and the Breusch–Pagan results confirmed the heteroscedasticity of residuals (χ^2^=92.49, *p*=0.0001). In such cases, it is suggested to use HC standard errors [18]. Next, a multiple regression with the same variables was run using the HC standard errors. The results showed that although the HC standard errors slightly lowered the coefficients for sex (*B*=−1.30, *SE* (HC)=0.55, *t*=−2.36, *p*=0.02), certainty of being infected (*B*=1.40, *SE* (HC)=0.29, *t*=4.78, *p*=0.0001) and anxiety (*B*=0.28, *SE* (HC)=0.05, *t*=4.77, *p*=0.0001), the variables still significantly predicted the DV. To test the homoscedasticity effects, a multiple regression with homoscedasticity-robust standard errors was run, and the results similarly showed that the sex, certainty of being infected and anxiety predicted the DV. The tolerance and the variance inflation (VIF) of included IVs showed no multicollinearity between IVs ((tolerance >0.20); tolerance range=0.44–1.0; (VIF ⩽10); VIF range=1.01–2.29), and the visual inspection of the scatter plot confirmed the linearity assumption.

Regarding the ANOVA of differences in COVID-like symptoms between age groups and across males and females, the variable age including five age groups (i.e. 0–20, 21–30, 31–40, 41–50 and 51–70) were entered into the analysis. The violation of normality was not a source of concern for this analysis, as ANOVA tests are known to be robust to non-normal distributions.

## Results

### Descriptive statistics

The means and ‘SDs’ of the study variables for males and females are shown in Table I. The distribution of symptoms across males and females is provided in Supplemental Material 1. Regarding the certainty of being infected, 26.9% of participants reported ‘sure not infected’, 45.6% reported ‘probably not infected’, 17.9% reported ‘uncertain’, 7.5% reported ‘quite certain’ and 2.1% reported ‘certain’ about being infected with COVID-19.

**Table I.** Descriptive statistics of the included variables.

Sex	Certainty	C-symptoms	Age	Anxiety	Stress	Ep	Ap	Cp	Np	Op
Females *M; SD N*=249 (min; max)	1.08; 0.89 (0; 4)	6.63; 5.16 (0; 24)	32.64; 10.24 (16; 67)	7.18; 7.40 (0; 34)	12.93; 9.30 (0; 38)	6.22; 1.97 (2; 10)	7.46; 1.58 (3; 10)	6.98; 1.89 (2; 10)	6.34; 2.19 (2; 10)	6.98; 1.89 (3; 10)
Males *M; SD N*=126 (min; max)	1.21; 1.07 (0; 4)	5.18; 5.34 (0; 24)	32.88; 12.13 (17; 79)	5.63; 6.67 (0; 38)	9.60; 8.27 (0; 40)	5.75; 1.91 (2; 10)	7.10; 1.75 (2; 10)	7.11; 1.77 (2; 10)	5.48; 2.26 (2; 10)	6.74; 1.68 (2; 10)
Total *M; SD N*=375 (min; max)	1.13; 0.96 (0; 4)	6.14; 5.26 (0; 24)	32.72; 10.90 (16; 79)	6.80; 7.35 (0; 38)	6.07; 1.96 (0; 40)	6.07; 1.96 (2; 10)	7.35; 1.64 (2; 10)	7.02; 1.86 (2; 10)	6.06; 2.25 (2; 10)	7.00; 1.70 (2; 10)

*SD*: standard deviation; Certainty: certainty of being infected with COVID-19; C-symptoms: report of COVID-like symptoms; Ep: extroversion; Ap: agreeableness; Cp: conscientiousness; Np: neuroticism; Op: openness personality dimensions.

### Correlations

Except age, extraversion, agreeableness, conscientiousness and openness, other study variables correlated with the reporting of COVID-like symptoms for both males and females (Table II).

**Table II.** Correlations between study variables across males and females.

	1	2	3	4	5	6	7	8	9	10
1. Age	1	−0.13	−0.08	−0.20**	−0.24**	−0.03	−0.01	0.21**	−0.14	0.22**
2. C-symptoms	0.01	1	0.26**	0.43**	0.49**	−0.03	−0.08	−0.01	0.30**	0.03
3. Certainty	0.05	0.42**	1	0.30**	0.25**	0.15	0.01	0.11	0.03	0.00
4. Stress	−0.20**	0.30**	0.13	1	0.76**	−0.02	−0.14	−0.12	0.42**	−0.01
5. Anxiety	−0.21**	0.43**	0.24**	0.73**	1	0.08	−0.11	−0.12	0.44**	−0.13
6. Extraversion	0.05	−0.07	−0.02	−0.03	−0.06	1	−0.08	0.22*	−0.12	−0.03
7. Agreeableness	0.02	−0.12	−0.02	−0.18**	−0.21**	0.16**	1	0.04	−0.24**	0.04
8. Conscientiousness	0.27**	−0.01	0.11	−0.11	−0.20**	0.12	0.20**	1	−0.21**	0.01
9. Neuroticism	−0.19**	0.13*	0.14*	0.54**	0.43**	−0.1	−0.25**	−0.20**	1	−0.05
10. Openness	0.02	−0.01	−0.00	0.12	0.04	0.05	0.15*	0.13*	0.09	1

Correlations for females are shown on the lower left side of the table (below the 1 correlations), and for males at the upper right side of the table (above the 1 correlations).

* *p*<0.05; **p<0.01.

C-symptoms: report of COVID-like symptoms.

### Moderated mediation analysis

#### Direct relationships

Certainty of being infected predicted the severity of reported COVID-like symptoms (*t*=3.26, *p*=0.001, *B*=2.84, *SE* (HC)=0.90). Moreover, anxiety (*t*=4.75, *p*=0.00001, *B*=0.26, *SE* (HC)=0.05) also predicted the severity of reported COVID-like symptoms. The direct effects of stress (*t*=0.77, *p*=0.44, *B*=0.03, *SE* (HC)=0.03), neuroticism (*t*=0.11, *p*=0.86, *B*=−0.05, *SE* (HC)=0.11) and sex (*t*=0.16, *p*=0.86, *B*=0.11, *SE* (HC)=0.66) were not significant.

#### Indirect relationships

The moderating indirect effect of sex on the effect of certainty of being infected on COVID-like symptoms was not significant (*t*=−1.90, *p*=0.057, *B*=−1.11, *SE* (HC)=0.58). The moderated mediation model predicted 29% of variance of severity of the COVID-like symptoms (Supplemental Material 2).

### Mediation analysis

**Figure 1.**
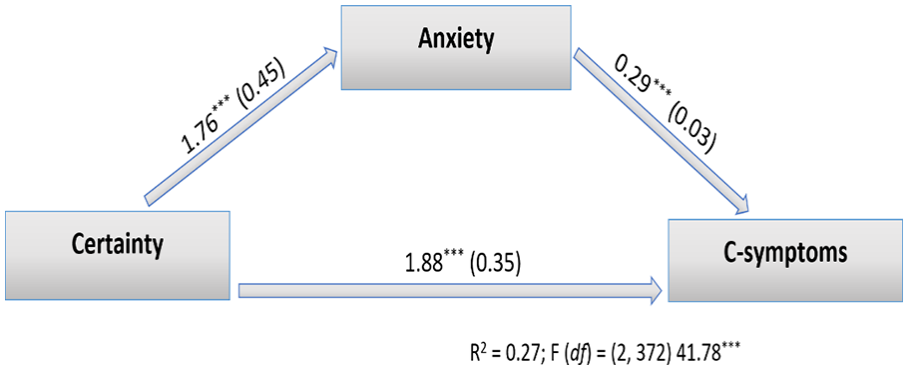
The accepted model with direct and indirect paths. Certainty of being infected directly predicted the dependent variable. Anxiety mediated the effects of certainty. The model explained 27% of variance in perceived severity of COVID-like symptoms. Certainty: certainty of being infected; C-symptoms: COVID-like symptoms. ***p<0.001.

The moderated mediation analysis showed that in addition to certainty of being infected, only anxiety predicted the reports of COVID-like symptoms. Next, whether anxiety mediated the effect of certainty of being infected on COVID-like symptoms was tested using the Sobel test. First, the DV, reporting of COVID-like symptoms, was regressed on the IV, certainty of being infected. The results showed that certainty of being infected predicted the report of COVID-like symptoms (*B*=1.88, *SE* (HC)=0.35, *t*=5.24, *p*=0.0001, *R*^2^=0.11). Then, the mediator, anxiety, was regressed on the IV, and the results showed that certainty of being infected predicted anxiety (*B*=1.76, *SE* (HC)=0.45, *t*=3.87, *p*=0.0001, *R*^2^=0.05). For the last step, the DV was regressed on both the IV and the mediator. The results showed that both certainty of being infected (*B*=1.36, *SE* (HC)=0.30, *t*=4.43, *p*=0.00001) and anxiety (*B*=0.29, *SE* (HC)=0.03, *t*=7.75, *p*=0.00001) predicted the severity of COVID-like symptoms (*R*^2^=0.27, *F*(2, 372)=41.78, *p*=0.0001; Sobel test statistics for anxiety using *SE* (HC)=4.43, *SE*=0.14, *p*=0.000001; Figure 1).

Finally, the modified version of the proposed model predicted 27% of the variance of severity of the COVID-like symptoms (Table III).

**Table III.** Mediation analysis results.

Predictors	Dependent variable	*B*	*SE* (HC)	*t*	*R* ^2^	*F* (*df*)
Step 1:						
Certainty	C-symptoms	1.88***	0.35	5.24	0.11	27.50*** (1, 373)
Step 2:						
Certainty	Anxiety	1.76***	0.45	3.87	0.05	15.02*** (1, 373)
Step 3:						
1. Certainty	C-symptoms	1.36***	0.30	4.43	0.27	41.78*** (2, 372)
2. Anxiety		0.29***	0.03	7.75		
Interaction:	C-symptoms	Sobel test^a^:				
Certainty×anxiety		4.43***	0.14	–	–	–

a To calculate the Sobel test, heteroscedasticity-consistent standard errors for certainty of being infected and anxiety were used.

*** p<0.001.

### Analyses of variance

The main effect of age (*F*(4, 365)=2.63, *p*=0.03, partial η^2^=0.03) was due to respondents in the age group 31–40 reporting higher COVID-like symptoms compared to the age groups 21–30 (mean difference=1.68, *SE*=0.68, *p*=0.01), 41–50 (mean difference=2.63, *SE*=1.05, *p*=0.01) and 51–79 (mean difference=2.48, *SE*=1.06, *p*=0.02).

The main effect of sex (*F*(1, 365)=7.47, *p*=0.007, partial η^2^=0.38) showed that females reported higher COVID-like symptoms compared to males (mean difference=2.08, *SE*=0.76, *p*=0.007; Supplemental Material 3). Females had higher anxiety (*F*(1, 373)=3.88, *p*=0.049, partial η^2^=0.01) and stress (*F*(1, 373)=11.51, *p*=0.001, partial η^2^=0.03) compared to males.

## Discussion

A model including certainty of being infected with coronavirus and anxiety predicted 27% of variance in the perceived severity of COVID-19 symptoms. Furthermore, a mediation analysis showed that the effect of certainty of being infected was partially mediated by anxiety, suggesting the effects of both variables on the reporting of COVID-like symptoms. Participant sex did not mediate or moderate these associations, but females had higher anxiety and stress levels and reported more COVID-like symptoms than males did.

Certainty of being infected with COVID-19 was a unique predictor of higher reports of COVID-like symptoms. In the present study, respondents who were more certain that they were or had been infected with the virus were more inclined to report symptoms. Prior studies have documented that expecting symptoms may lead to experiencing them. Cocco [19] showed that individuals who were fully informed about the sexual side effects of a treatment reported similar symptoms three times more than those in the control group who were not informed about the sexual side effects. It is not known, however, whether the association of certainty and symptoms was due to the participants attributing their bodily symptoms as related to COVID-19.

The association of certainty of being infected with the report of COVID-like symptoms was not moderated by the participants’ sex, which might be partially due to the lower number of males (*N*=126) than females (*N*=249) and low reports of symptoms in both males (*M*=5.26) and females (*M*=6.64). Nonetheless, females had higher reports of COVID-like symptoms compared to males. The higher reports of COVID-like symptoms by females are in line with the review by Vambheim and Flaten [12] that showed more nocebo effects in females. This finding suggests that females might be more prone to attribute bodily symptoms to COVID-19 symptoms, which is in line with the ‘symptom perception model’ put forth by Van Wijk and Kolk [20]. According to this model, females may attribute their physiological symptoms to health issues more than males do, partially due to social norms and socialisation processes, where females are better informed about health and well-being issues compared to males [20]. However, it should be noted that one study showed that participant sex was not a significant predictor of fear of COVID-19 [21]. Moreover, the higher reports of COVID-like symptoms in females can be partially explained by females’ higher levels of anxiety and stress. Lyby et al. [22] showed that the lower placebo effects in females could be explained by higher stress levels in females. Along the same line, the present study shows that higher anxiety predicts more COVID-like symptoms and also partly mediates the association of certainty of being infected and reporting COVID-like symptoms. Hence, the main effect of sex on higher COVID-like symptoms is due to the higher anxiety and stress levels in females and probably not to the female sex. This explanation is supported by the results of the moderated mediation analyses that showed that sex did not moderate the effects of certainty of being infected on the reporting of COVID-like symptoms, but anxiety did.

Regarding the role of negative emotions on the reporting of bodily sensations as COVID-19 symptoms, the results showed that anxiety was uniquely associated with reported COVID-like symptoms. Former research has shown that individuals who have higher negative emotions tend to report higher symptoms and treatment side effects. Cameron et al. [23] showed that for patients who underwent chemotherapy for breast cancer, those with higher anxiety had an increased tendency to attribute their symptoms to the chemotherapy compared to patients with lower anxiety. Therefore, it is possible that negative emotions such as anxiety triggered individuals’ attentiveness and sensitivity over their bodily experiences and consequently resulted in a higher perception of physiological experiences as COVID-19 symptoms.

Anxiety also partially mediated the association of certainty of being infected with the severity of reported COVID-like symptoms, as the mediation analysis showed that both certainty and anxiety predicted the COVID-like symptoms. The significant Sobel test showed that including anxiety as a mediator significantly reduced the effect of certainty of being infected on reported symptoms. Thus, a causal model is supported, where certainty of being infected leads to increased reporting of COVID-like symptoms. However, part of the effect of certainty is due to higher anxiety, suggesting that a higher certainty of being infected was associated with more reported COVID-like symptoms if the individual reported higher anxiety levels. Thus, reporting COVID-like symptoms is enhanced when individuals believe they have been infected or if the individuals are anxious. These cognitive and emotional factors have independent effects on symptom reporting. However, anxiety also has a mediating effect on the effect of certainty. Thus, the nocebo effect seems to consist of both cognitive and emotional components. Such a trend is in agreement with prior studies showing that negative emotions and thoughts (e.g. health-related concerns) result in over-reporting of symptoms [24]. This expands the model proposed by Colloca and Benedetti [25], where anxiety was identified as the main contributor to nocebo effects, by suggesting that cognitive factors may have an independent role. However, as the present study is a cross-sectional survey study, causality cannot be concluded from the results, and the suggested model should be followed up by experimental studies.

In the present study, neuroticism failed to be significantly associated with COVID-like symptoms and significantly mediating the relationship between certainty of being infected and COVID-like symptoms. Thus, the personality of the person believing to be or having been infected seems to be of little importance.

Finally, the moderated mediation analysis showed that participant age was not associated with the report of COVID-like symptoms, even though severity of symptoms is linked to higher age [3]. However, the trend analysis showed that participants aged between 31 and 40 years reported more symptoms than other older age groups. This is in line with studies that have suggested an opposite trend for age, as being younger was shown to be related with more psychological distress during COVID-19 pandemic [26]. However, the effect of age in this study might be affected by the age range of the participants, who had a mean of about 33 years, and the low report of symptoms. Thus, the present study is not conclusive about the effects of age.

## Conclusions

Believing to be infected with COVID-19, along with anxiety, can enhance the attribution of common bodily experiences to COVID-19. A theoretical model where the effect of certainty of being infected on COVID-like symptoms was partially mediated by anxiety was supported, suggesting the contribution of both cognitive and emotional factors on the manifestation of nocebo effects.

## Recommendations for future studies

Future studies are recommended to consider the following. First, as the design of the study was cross-sectional, causality cannot be drawn from present findings. Hence, future studies are recommended to test the model approved here through experimental studies. Second, certainty of being infected with COVID-19 could be modulated or mediated by other cognitive or personality factors. In a consecutive study, we showed that conscientiousness and health anxiety predicted the certainty of being infected with COVID-19 [27]. However, there might be other psychological constructs, for example health catastrophising (i.e. over-estimation of the health threats) that could also contribute to such a nocebo belief. Therefore, possible relationships between other cognitive factors such as catastrophising and attribution of bodily experiences as COVID-19 symptoms should be investigated in the future. Third, the effects of other negative emotions, such as depression, on attribution of bodily experiences as COVID-19 symptoms should be considered. Fourth, the present study was conducted using a sample from the general population. This may have affected the data in terms of low report of symptoms. Therefore, predictive values of negative emotions and cognitive factors require revalidation in clinical populations as well. Fifth, the effects of contextual factors, such as exposure to constant news about the pandemic [8], or the behaviour and characteristics of health-care personnel [28] on attentiveness to bodily sensations and attributing them to COVID-19 should be investigated. Lastly, previous studies have shown that placebo/nocebo effects modulate physiological activity such as the cardiac system [29]. Thus, experimental studies are encouraged to investigate the effects of nocebo factors on the exacerbation of physical symptoms in COVID-19 positive patients, as in Lyby et al. [22].

## Limitations

The present study was conducted using the general population, which probably led to low reporting of symptoms and thus lower variability. This might have affected the results. There were also relatively more females than males which might have affected the results. Future studies are recommended to control for these issues. Regarding the cross-sectional design of the study, causation could not be implied from the results. Moreover, online surveys have a number of problems such as biases in the sampling process, the effects of fatigue and loss of interest during responding to the questions, and dishonest responses. However, to lower the fatigue effect, only scales and questions that were considered easy to comprehend and respond were included. The number of questions was also kept at a minimum (*N*=57) to ensure that filling out the whole survey would not take more than 12 minutes. To avoid biased sampling, the link to the survey was manually distributed on social media such Facebook, Instagram and Twitter. However, as the descriptive results showed, the majority of the respondents were highly educated. So, caution should be paid in generalising the present findings to other populations. The present study also did not check for other underlying health issues due to the limitations in the number of questions. Chronic health issues may sensitise individuals to their bodily experiences [30]. Therefore, future studies should also investigate the effects of underlying health issues on attribution of bodily experiences to coronavirus symptoms. Lastly, this study only tested respondents who reported as not having tested positive for COVID-19. However, there is still a possibility that some of the respondents were infected without knowing it.

## Supplemental Material

sj-docx-1-sjp-10.1177_14034948211018385 – Supplemental material for Experiencing COVID-19 symptoms without the disease: The role of nocebo in reporting of symptoms Click here for additional data file. Supplemental material, sj-docx-1-sjp-10.1177_14034948211018385 for Experiencing COVID-19 symptoms without the disease: The role of nocebo in reporting of symptoms by Hojjat Daniali and Magne Arve Flaten in Scandinavian Journal of Public Health

sj-docx-2-sjp-10.1177_14034948211018385 – Supplemental material for Experiencing COVID-19 symptoms without the disease: The role of nocebo in reporting of symptoms Click here for additional data file. Supplemental material, sj-docx-2-sjp-10.1177_14034948211018385 for Experiencing COVID-19 symptoms without the disease: The role of nocebo in reporting of symptoms by Hojjat Daniali and Magne Arve Flaten in Scandinavian Journal of Public Health

sj-docx-3-sjp-10.1177_14034948211018385 – Supplemental material for Experiencing COVID-19 symptoms without the disease: The role of nocebo in reporting of symptoms Click here for additional data file. Supplemental material, sj-docx-3-sjp-10.1177_14034948211018385 for Experiencing COVID-19 symptoms without the disease: The role of nocebo in reporting of symptoms by Hojjat Daniali and Magne Arve Flaten in Scandinavian Journal of Public Health
