# Supplementary material for: Experiencing COVID-19 symptoms without the disease: The role of nocebo in reporting of symptoms
Source: Scand J Public Health. 2021 May 27;50(1):61–9. doi: 10.1177/14034948211018385 (PMC8807543; doi:10.1177/14034948211018385)
Supplement: sj-docx-1-sjp-10.1177_14034948211018385 – Supplemental material for Experiencing COVID-19 symptoms without the disease: The role of nocebo in reporting of symptoms [file sj-docx-1-sjp-10.1177_14034948211018385.docx]

| C-symptoms | Sex N (%) | None  (0) | Very mild (1) | Mild  (2) | Moderate (3) | Severe  (4) |
| --- | --- | --- | --- | --- | --- | --- |
| Headache | Males | 48 (38.1) | 30 (23.8) | 28 (22.2) | 16 (12.7) | 4 (3.2) |
|  | Females | 47 (18.9) | 69 (27.7) | 62 (24.9) | 56 (22.5) | 15 (6.0) |
| Bodily pain | Males | 79 (62.7) | 27 (21.4) | 9 (7.1) | 9 (7.1) | 2 (1.6) |
|  | Females | 120 (48.2) | 48 (19.3) | 45 (18.1) | 30 (12.0) | 6 (2.4) |
| Chills | Males | 93 (73.8) | 17 (13.5) | 11 (8.7) | 4 (3.2) | 1 (0.8) |
|  | Females | 172 (69.1) | 46 (18.5) | 21 (8.4) | 9 (3.6) | 1 (0.4) |
| Fever | Males | 102 (81.0) | 14 (11.1) | 4 (3.2) | 4 (3.2) | 2 (1.6) |
|  | Females | 205 (82.3) | 22 (8.8) | 9 (3.6) | 9 (3.6) | 4 (1.6) |
| Persistent fever | Males | 119 (94.4) | 3 (2.4) | 2 (1.6) | 1 (0.8) | 1 (0.8) |
|  | Females | 235 (94.4) | 4 (1.6) | 6 (2.4) | 2 (0.8) | 2 (0.8) |
| Repeated coughing | Males | 82 (65.1) | 31 (24.6) | 9 (7.1) | 2 (1.6) | 2 (1.6) |
|  | Females | 182 (73.1) | 41 (16.5) | 14 (5.6) | 11 (4.4) | 1 (0.4) |
| Breathing difficulty | Males | 101 (80.2) | 14 (11.1) | 6 (7.1) | 4 (3.2) | 1 (0.8) |
|  | Females | 197 (79.1) | 31 (12.4) | 11 (4.4) | 5 (2.0) | 5 (2.0) |
| Sore throat | Males | 70 (55.0) | 33 (26.2) | 14 (11.1) | 7 (5.6) | 2 (1.6) |
|  | Females | 132 (53.0) | 69 (27.7) | 25 (10.0) | 20 (8.0) | 3 (1.2) |
| Fatigue | Males | 57 (45.2) | 30 (23.8) | 24 (19.0) | 11 (8.7) | 4 (3.2) |
|  | Females | 69 (27.7) | 71 (28.5) | 47 (18.9) | 48 (19.3) | 14 (5.6) |
| Fever/Dry cough/ Short breath | Males | 119 (94.4) | 4 (3.2) | 1 (0.8) | 0 | 2 (1.6) |
|  | Females | 237 (95.2) | 7 (2.8) | 3 (1.2) | 1 (0.4) | 1 (0.4) |

**Supplementary Material 1**

*Distribution of Coronavirus-like Symptoms Across Males and Females*

*Note*. C-symptoms: report of COVID-like symptoms.
