# Supplementary material for: Experiencing COVID-19 symptoms without the disease: The role of nocebo in reporting of symptoms
Source: Scand J Public Health. 2021 May 27;50(1):61–9. doi: 10.1177/14034948211018385 (PMC8807543; doi:10.1177/14034948211018385)
Supplement: sj-docx-2-sjp-10.1177_14034948211018385 – Supplemental material for Experiencing COVID-19 symptoms without the disease: The role of nocebo in reporting of symptoms [file sj-docx-2-sjp-10.1177_14034948211018385.docx]

**Supplementary material 2**

*The Moderated Mediation Analysis Results*

| Predictors | *B* | *S.E* (HC) | *t* |
| --- | --- | --- | --- |
| Certainty | 2.84^***^ | 0.90 | 3.26 |
| Stress | 0.03 | 0.03 | 0.77 |
| Anxiety | 0.26^***^ | 0.05 | 4.75 |
| Neuroticism | -0.05 | 0.11 | 0.11 |
| Sex | 0.11 | 0.66 | 0.16 |
| Certainty*Sex | -1.11 | 0.58 | -1.90 |
|  |  |  |  |
| *R*^2^ (*MSE*) | 0.29 (19.86) |  |  |
| *F*; (*df*) | 17.99^***^; (6, 368) |  |  |

*Note*. Dependent variable: COVID-like symptoms. B: coefficients. *S.E* (HC): heteroscedasticity-consistent standard error. *MSE*: mean standard error. * *p* < .05; ** *p* < .01; *** *p* < .001.
