# Supplementary material for: Experiencing COVID-19 symptoms without the disease: The role of nocebo in reporting of symptoms
Source: Scand J Public Health. 2021 May 27;50(1):61–9. doi: 10.1177/14034948211018385 (PMC8807543; doi:10.1177/14034948211018385)
Supplement: sj-docx-3-sjp-10.1177_14034948211018385 – Supplemental material for Experiencing COVID-19 symptoms without the disease: The role of nocebo in reporting of symptoms [file sj-docx-3-sjp-10.1177_14034948211018385.docx]

**Supplementary Material 3**


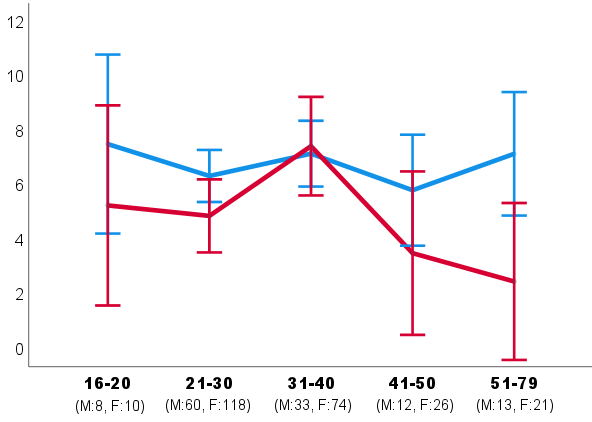
 *Reporting of COVID-like Symptoms in Age Groups Across Males and Females*

*Note*. The lines show the estimated marginal means for the reports of COVID-like symptoms (vertical axis) in the five age groups (horizontal axis) across males (red line) and females (blue line). Respondents in the age group 31-40 reported higher COVID-like symptoms compared to the age groups 21-30, 41-50, and 51-79. Moreover, females reported higher COVID-like symptoms compared to males. Numbers in parentheses below age groups are *Ns* for males (M) and females (F).

Error bars: +/- 2 *SE*.
